# A Taxonomy of Six Perceptual Cues Underlying Photorealism in 3D-Rendered Architectural Scenes: A Cue-Based Narrative Review

**DOI:** 10.3390/jimaging12030113

**Published:** 2026-03-08

**Authors:** Matija Grašić, Andrija Bernik, Vladimir Cviljušac

**Affiliations:** 1 Department of Computer Graphics and Multimedia Systems, Faculty of Graphic Arts, University of Zagreb, 10000 Zagreb, Croatia; mgrasic@unin.hr (M.G.); vladimir.cviljusac@grf.unizg.hr (V.C.); 2 Department of Multimedia, University North, 42000 Varaždin, Croatia

**Keywords:** photorealism, architectural visualization, visual perception, global illumination, contact shadows, physically based rendering

## Abstract

Perceived photorealism in architectural 3D rendering is not determined solely by physical accuracy or rendering complexity but also by a limited set of visual cues that observers rely on when judging realism. This literature review synthesizes findings from 41 peer-reviewed studies spanning perception science, computer graphics, and immersive visualization, with the aim of identifying the cues that most strongly contribute to perceived photorealism in rendered scenes. Convergent evidence from psychophysical experiments, user studies in virtual and augmented reality, and rendering-oriented analyses indicate that six cue categories consistently dominate realism judgments. Across the reviewed literature, realism judgments depend less on scene complexity or the number of visual elements and more on the consistency and plausibility of these cues for supporting inferences about shape, material, and spatial layout. These findings suggest that photorealism emerges from the alignment of the rendered image structure with perceptual expectations learned from real-world visual experience. The implications for architectural visualization workflows and directions for future research on cue interactions and perceptual thresholds are discussed.

## 1. Introduction

Architectural photorealism is often framed as the outcome of physically based rendering, detailed assets, and accurate light transport. However, observers do not judge photorealism by estimating physical accuracy; they rely on a limited set of diagnostic visual cues when inferring shape, material, and spatial relationships [1,2]. In this paper, a visual cue is defined as any image-level feature or regularity that observers use to infer spatial layout, material properties, surface form, or overall plausibility when judging whether a rendered scene appears realistic. Accordingly, we treat photorealism as perceptual plausibility: the extent to which rendered image structure matches learned regularities from real-world vision.

A substantial body of research has shown that both presence (i.e., the sense of being immersed) and plausibility (how convincing an experience appears) in immersive spaces are highly correlated with the degree to which observers can interpret the scene as visually coherent in light and shadow [3]. Higher visual detail can increase engagement but also raises cognitive load when it is inconsistent across the scene; detail alone does not guarantee higher perceived realism [4].

Beginning with empirical studies in the field of computer graphics perception, many researchers have confirmed that some visual cues are much more influential in determining how realistic a viewer perceives a scene to be. One classic series of experiments has shown that certain visual features, such as contact shadows and surface imperfections, can determine how viewers classify a scene as either real or not real, regardless of changes in scene complexity or the number of objects in the scene [5]. The findings from these types of studies support perceptual evidence that demonstrates an object’s ability to produce a cast shadow is a highly reliable indicator of both the contact between an object and its environment and the spatial relationships between multiple objects [6] and that shadow geometry contains structured information regarding the direction of the light source and the shape/form of surfaces [7]. Furthermore, research on spatial perception in rendered environments (i.e., computer-generated images (CGIs)) suggests that lighting/shadow visual cues frequently outweigh all other pictorial visual cues in terms of supporting viewers’ accurate perceptions of spatial relationships between objects within a scene [8,9].

These visual cues continue to play important roles in immersive and augmented environments as well, where the rendered content must visually interact/combine with the physical or virtual surroundings of the environment. In both virtual and augmented reality settings, studies have demonstrated that adding plausible cast shadows and a coherent lighting scheme greatly enhances viewers’ accuracy in estimating distances and understanding the spatial arrangement of virtual objects [10,11,12].

Moreover, material appearance research highlights the contributions of surface texture, reflectance characteristics, and small-scale surface irregularities to viewers’ perceptions of realism. Research using image statistics-based approaches has revealed that viewers are responsive to specific diagnostic texture and reflectance signatures and that subtle micro-imperfections and view-dependent effects such as specular reflection significantly contribute to the perceived realism of materials [13,14,15].

Existing reviews from related fields, such as perception of material, realism of rendering, presence in immersion, and rendering technique, have identified some of the same visual cue issues; however, they have rarely synthesized that evidence into a cue-based synthesis focused on architectural 3D renderings. Therefore, while the contribution of this review is a compilation of previous research results, its value lies in integrating those results into a six-category perceptual taxonomy to identify which visual cues are most consistent in influencing realism and how those cues can be used to determine priorities in rendering in architectural visualization.

## 2. Conceptual Background and Cue Categories

This section defines the six cue categories used as the organizing framework for the literature review. It establishes the conceptual vocabulary for the synthesis that follows, but does not assess the relative weight of each category. Comparative analysis of cue strength, threshold behavior, and workflow relevance is addressed in Section 4.

### 2.1. Global Illumination and Indirect Lighting

Global illumination (GI) refers to indirect light transport effects such as interreflection, diffuse bounce light, and soft indirect shadowing, which contribute to the perception of shape and spatial organization in a scene [8]. From a rendering standpoint, photon-based methods have been shown to provide efficient approximations of these effects [16]. Several studies of indirect illumination have also examined detection thresholds for perceptible differences using visibility models and perceptually based error metrics [17,18]. In this review, GI is treated as a fundamental category of lighting-related cues, while its relative impact on realism judgments is discussed in Section 4 [10].

### 2.2. Contact Shadows and Penumbra Behavior

Contact shadows and penumbra behavior provide cues about object grounding, physical contact, relative depth, and spatial arrangement [6]. The geometry and structure of shadow edges also convey information about surface shape and light source position [7]. Research in computer graphics perception has examined the visual characteristics of shadows, including softness and penumbra structure, in the context of realism judgments [5,19], and has also explored efficient rendering techniques for reproducing these effects [20]. In this review, the role of shadows and shadow characteristics across different display conditions, including reduced-detail virtual and augmented reality environments, is synthesized in Section 4 [11,12,21].

### 2.3. Texture Resolution and Physically Based Material Representation

Texture resolution and physically based material representation contribute to material appearance by providing surface detail and supporting consistent reflectance behavior. Research in interactive rendering and user-annotated datasets has examined how observers perceive reductions in texture fidelity relative to other forms of visual degradation, such as geometric simplification [22]. Related work in VR-based digital twins has also investigated how texture and geometric detail influence perceived realism and quality [23]. Psychophysical studies further show that observers can use statistical texture information even when fine surface details are near or just above display resolution [24], while predictive models have examined texture degradation as a visible rendering artifact [25]. The relative perceptual importance of these effects is synthesized in Section 4.

### 2.4. View-Dependent Material Cues

View-dependent effects include specular reflections, highlight structure, and Fresnel modulation, all of which vary with viewing direction and influence perceived material appearance and shape perception. Early psychophysical studies identified perceptually meaningful dimensions of gloss based on highlight properties [15], and subsequent work showed that the geometry of specular reflections also affects shape perception [26]. Further research has examined how observers use specular image structure to make material inferences and which variations in specular and diffuse appearance are perceptually significant [27,28]. Fresnel-related changes in reflectance and other viewpoint-dependent image effects have also been studied in relation to gloss stability, realism, and presence in virtual environments [29,30]. The relative importance of these effects is discussed in Section 4.

### 2.5. Micro-Imperfections and Surface Roughness

Micro-imperfections and surface roughness refer to small-scale surface irregularities that influence the appearance of realistic materials. There is evidence that such surface variation is important for the perception of realism [5], and vision research has shown that statistical image properties can be used to infer surface characteristics from images [13]. Studies have also examined the relationship between perceived roughness and gloss, including the effects of inconsistencies between roughness and reflectance [14], as well as the contribution of learned image regularities to gloss perception [31]. In rendering practice, some of these effects appear as glints, sparkles, and fine-scale surface variation, and several studies have examined their visual and material significance [32,33,34]. Their place within the broader cue hierarchy is discussed in Section 4.

### 2.6. Subsurface Scattering and Translucency

Subsurface scattering and translucency represent a specific category of material cues based on light transport through partially light-permeable materials. Studies have shown that translucency perception depends strongly on image-level cues, such as edge behavior and luminance variation across material thickness, and less directly on the physical properties of the material itself [35]. Psychophysical studies have also examined how translucency affects shape perception and how these effects can be described using low-dimensional perceptual models [36,37]. Additional work has investigated the influence of lighting direction, as well as the relative importance of edges and thin features in translucency judgments [38,39]. These effects are treated here as context-dependent material cues, and their perceptual implications are synthesized in Section 4.

## 3. Methodology and Research Process

**Figure 1:**
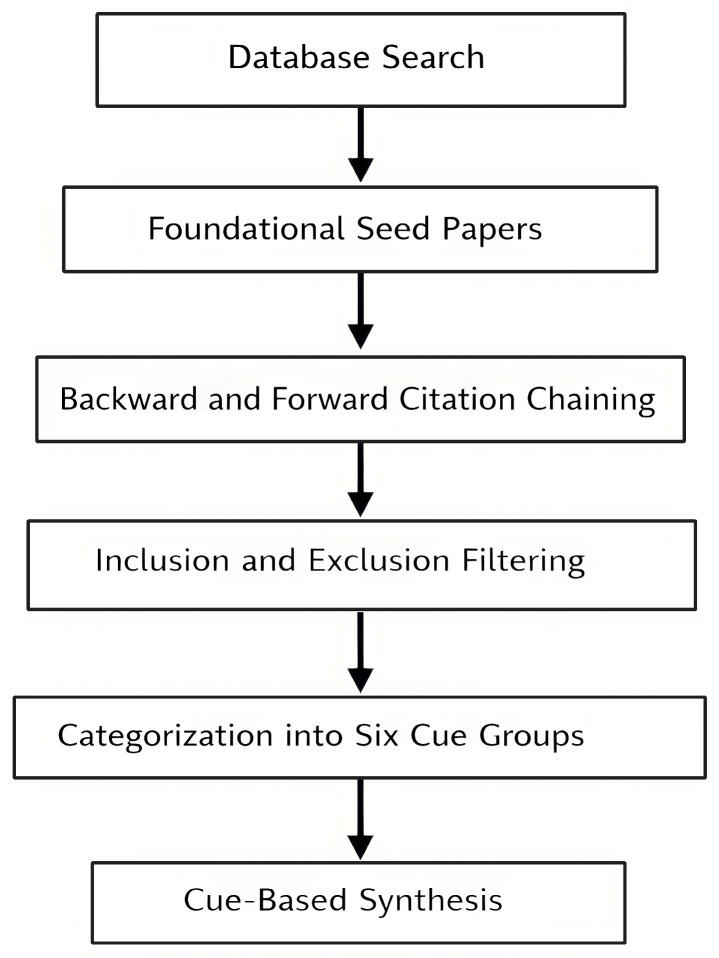
Overview of the review methodology, including source identification, selection, categorization, and cue-based synthesis.

The research described here is a visual cue-based, narrative literature review of a predetermined set of 41 peer-reviewed articles that examined the relationship between visual cues and perceived photorealism of architectural 3D renderings. The study focused on those visual cues present at an image level that result in higher degrees of realism and/or material plausibility in addition to those resulting in greater spatial coherence in rendered images. The main steps of the review methodology, from source identification to cue-based synthesis, are summarized in Figure 1.

This review uses a visual cue-based narrative synthesis grounded in perceptual theory, rather than a PRISMA-style systematic review. Here, “PRISMA-style systematic review” refers to a formal review process based on structured search, screening, and reporting procedures designed to support transparent study selection and reproducibility. We distinguish the present work from that format because the reviewed studies vary substantially in stimuli, tasks, and outcome measures, making a cue-based narrative synthesis more appropriate than a pooled systematic comparison. Because stimuli, tasks, and outcome measures vary widely across vision science, computer graphics, and immersive visualization, we compare convergent findings within each cue category instead of pooling results across all studies.

To identify relevant research, a focused search in top academic online databases (ACM Digital Library, IEEE Xplore, SpringerLink, and The Journal of Vision) was performed. Well-known research that provided foundational knowledge of material perception, light and shadow analysis, and perceptual realism formed an initial set of references from which backward and forward citation searching was conducted to expand this list.

### 3.1. Inclusion Criteria and Categorization

The corpus of the reviewed articles was compiled by way of a theory-based, targeted process that encompassed three areas of research: (1) scientific studies on the perception of light, materials and spatial cues; (2) computer graphics and rendering research with an emphasis on perceptual realism; and (3) studies conducted in virtual reality (VR) and augmented reality (AR) to assess realism-related visual cues in immersive environments. The scope of this review was intentionally limited to studies examining perceptual realism through interpretable image-level cues in rendered images, rather than to recent neural rendering pipelines treated primarily as generative or reconstruction frameworks.

To be considered within this review, a study needed to meet at least one of the following three (3) inclusion criteria:

– The study examined empirically one or more of the visual cues that are associated with realism (i.e., material appearance, lighting, spatial perception).
– The study included human subjects via either psychophysical experimentation, user study in Virtual Reality/Augmented Reality, or evaluation of rendered images based upon a perceptual motivation.
– The results of the study provided some evidence concerning the degree to which rendered images appeared realistic, plausible, believable as material, or interpretable in terms of spatial relationships.

Studies were excluded if they:

– Were not published through a peer-review process.
– Examined only the performance of an algorithm (and did so without providing any information regarding how such performance could potentially relate to perceptions of what is being rendered).
– Only addressed the concept of realism in terms of physical accuracy and did not provide information about how observers perceive the image.
– Focused primarily on neural rendering or AI-based scene synthesis methods (e.g., NeRF-based or Gaussian Splatting approaches) unless they provided direct empirical evidence about specific image-level visual cues relevant to human judgments of realism.

Each study was categorized based on the primary visual cue(s) it examined based on the extraction of specific cues that were determined to be relevant across the reviewed literature. The process resulted in several recurrent themes that allowed the literature to be categorized into six distinct cue categories: global illumination and indirect lighting; contact shadows and penumbra behavior; texture resolution and physically based representations of materials; view-dependent effects; micro-imperfections and surface roughness; and subsurface scattering and translucency.

### 3.2. Synthesis Strategy

Rather than summarizing all the research that has been done in a sequence to provide an overall view of the findings of all the studies reviewed, this review uses a cue-based synthesis method to compare the findings of the studies for each cue category (i.e., whether the findings from the various studies reviewed are similar or dissimilar) across the experimental task (e.g., visual search), type of stimulus (e.g., images vs. videos), population of observers and perceived effects of the cues. This review places greater weight on empirical evidence than on evidence of how algorithms work, but it includes algorithmic studies when these studies provide some insight into the behavioral characteristics of the cues relative to perception.

### 3.3. Treatment of Realism as a Perceptual Construct

In addition to the different ways in which photorealism can be defined across the reviewed literature (including binary realism judgments; subjective quality ratings; measures of spatial or material perception; and measures of presence or plausibility for users within an immersive environment), the authors treat realism as a family of perceptual results on the basis of shared visual cues that support these results. The authors’ view aligns with theoretical models suggesting that visual realism occurs because image statistics match the perceptual expectations derived from real-world experiences rather than because of physical correctness [1,2].

### 3.4. Methodological Limitations

This review considers non-visual contributing factors (e.g., semantic plausibility, task knowledge) only when they directly interact with visually identifiable cues. Additionally, most seminal psychophysics studies have used relatively simple stimuli and small samples of subjects to facilitate statistical analysis and data collection and thus may have low ecological validity. Conversely, VR and AR studies typically use higher levels of realism but less control over the experiments. These trade-offs should be understood as an important methodological limitation of the reviewed literature; however, the convergence of findings across controlled psychophysical studies and more ecologically realistic VR/AR studies still strengthens confidence in the overall cue-based interpretation.

## 4. Results and Synthesis

**Figure 2:**
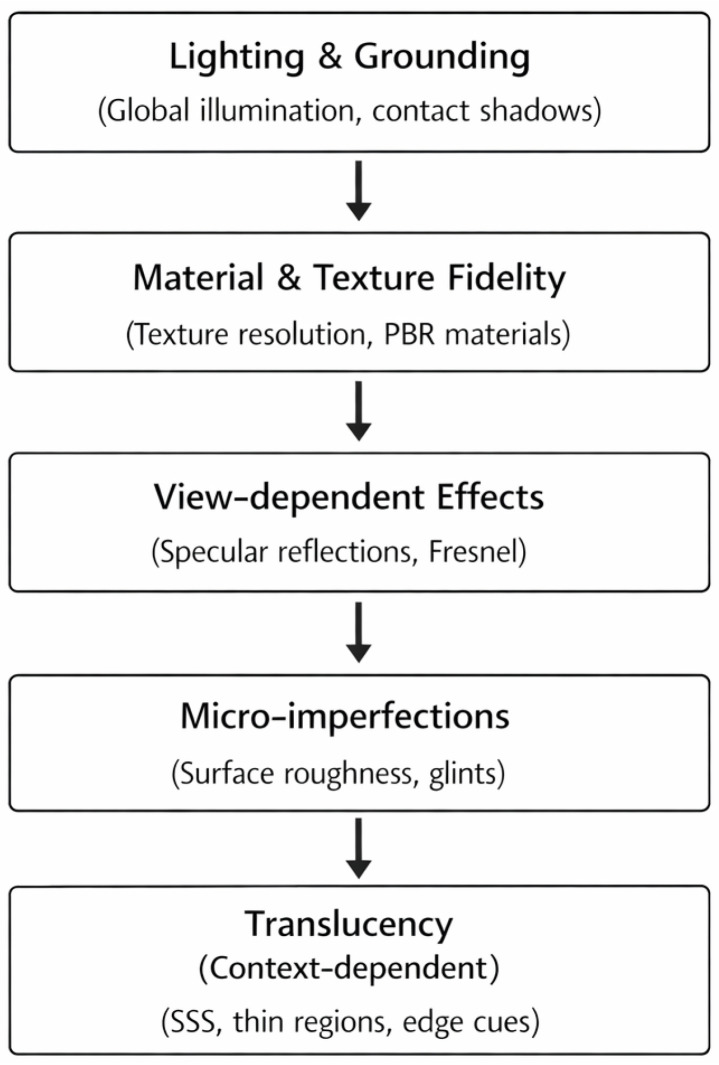
Author-generated schematic illustration of the five hierarchical levels derived from the six visual cue categories discussed in this review.

This section synthesizes the reviewed evidence across the six cue categories introduced in Section 2 and reorganizes them into the five hierarchical levels shown in Figure 2. Rather than re-defining the cues, it evaluates their relative perceptual importance, context dependence, and practical implications for architectural visualization. Figure 2 summarizes the resulting hierarchy based on (I) consistency of evidence across studies and (II) reported impact on realism judgments. This ordering is not intended to represent a temporal sequence of perception (i.e., not the order in which viewers literally “see” scene properties), but rather an analytical hierarchy derived from the convergence of findings across the reviewed studies. In other words, the order reflects relative explanatory importance within the literature, not a fixed moment-by-moment perceptual timeline. An overview of the dominant perceptual cue categories, their primary perceptual functions, reported effects, and representative studies is provided in Table 1.

### 4.1. Lighting and Grounding

Lighting and grounding cues form the first and most influential level of the hierarchy shown in Figure 2. Across study paradigms, shadow presence and shadow plausibility emerge as gatekeeper cues for realism judgments: missing or implausible shadows (e.g., unnaturally sharp contact regions) consistently reduce “real” judgments. This sensitivity is consistent with evidence that shadows constrain perceived contact, relative depth, light direction, and surface form [6,7].

Global illumination complements this role by stabilizing luminance gradients, supporting interreflection, and increasing scene coherence, which together improve the readability of shape and spatial relations [8]. When rendering resources are limited and users are forced to make difficult choices, users in immersive VR environments have shown that they consistently choose to prioritize global illumination over other visual elements when deciding which features should be rendered first [10]. Studies on early spatial perception further revealed that while multiple pictorial cues can work together to create an accurate sense of space, cast shadows repeatedly emerged as one of the most important cues for determining an object’s position and size in relation to other objects in the environment. In match-to-sample positioning tasks, the addition of cast shadows improved positioning accuracy by 45.2%, whereas adding linearity improved positioning accuracy by 28.9%, indicating the importance of shadow cues in helping viewers determine relative positions [9].

Across these cue types, the largest perceptual gains typically occur once visual behavior becomes plausible; beyond that threshold, further increases in physical accuracy often yield diminishing returns. This threshold behavior is especially clear in the case of shadows. Comparative studies of soft-shadow algorithms show that reproducing qualitatively plausible penumbra behavior (e.g., contact hardening) is often sufficient to satisfy observer expectations, particularly when no direct reference is available [19]. Practical implementations such as PCSS (Percentage-Closer Soft Shadows) [20] are consistent with this perceptual logic. In AR, a similar threshold logic applies to distance perception: a significant improvement in distance estimation occurs when a single, coherent cast shadow is present, whereas detailed differences in shadow softness or shadow shape are secondary to providing a consistent shadow cue and coherent lighting direction [11,12].

Similarly, for global illumination, comparable threshold behavior is observed: qualitatively plausible interreflection and indirect shadow structure can be achieved with photon mapping and related approaches without requiring fully exhaustive physical simulation [16]. Approaches to perceptual engineering provide formalized measures for determining when differences in illumination become observable and therefore identify detectability thresholds beyond which additional sampling and/or physically correct light transport will not contribute meaningfully to perceived realism [17,18]. Research also shows that shadow plausibility becomes more important as geometric detail decreases, in part because it helps maintain a stable framework for interpreting space. At extremely low levels of geometry (e.g., 10% level of detail), shadow plausibility can become the primary indicator of perceived realism [21].

### 4.2. Material and Texture Fidelity

The second level of the hierarchy concerns material and texture fidelity, which builds upon the lighting and grounding framework established by the first level. Once spatial plausibility has been established, realism increasingly depends on whether surface appearance and reflectance behavior remain materially believable.

Studies across multiple paradigms show that both texture fidelity and physically based material representations perform better than the amount of detail in the underlying model geometry in predicting perceived realism and/or quality. Large-scale paired comparisons indicate that users typically notice texture degradation more readily than geometry simplifications at the same level of detail, and that lighting implementation influences which errors are most visible [22]. In the context of VR inspection and digital twins, increasing texture resolution has been shown to improve users’ perception of realism and quality, whereas increasing geometric detail has relatively little effect on their assessment of overall simulation quality [23]. At the same time, psychophysical evidence shows that users can perceive fine surface structure even when the individual texture elements themselves are not fully resolvable, which suggests that the visual system relies on statistical surface information rather than only on explicit geometric resolution [24].

Taken together, these findings support the placement of material and texture fidelity as the second level in Figure 2: once the scene is spatially coherent, the believability of surfaces and materials becomes the next dominant source of realism judgments.

### 4.3. View-Dependent Effects

The third level of the hierarchy consists of view-dependent effects. These cues enhance realism in two complementary ways: they support inferences about material properties and shape, and they also function as authenticity signals during motion and interactive viewing.

Research on reflectance and material perception has shown that perceived gloss can be explained through a small number of perceptual parameters, including highlight contrast and sharpness, thereby providing rendering control with perceptually meaningful cue-related parameters [15]. The structure of specular reflection also supports shape perception by producing systematic image distortions related to surface orientation [26], and observers are able to categorize materials on the basis of specular image structure even when the observed shape is complex [27]. Additional work shows that both specular and diffuse appearance vary along a limited number of perceptually significant dimensions, which helps explain why some common reflection errors are especially noticeable [28].

Within this framework, Fresnel modulation is especially important because it is inherently view-dependent. Experimental findings show that Fresnel effects increase the perceived realism of gloss and promote gloss constancy across changing viewing and lighting conditions [29]. In immersive virtual reality environments built from multicamera capture, the inclusion of view-dependent image-based effects increases perceived realism and presence even when only minor visual artifacts are present, suggesting that viewpoint contingency itself acts as a strong signal of visual authenticity [30]. This aligns with broader definitions of presence in which coherence and plausibility are central to users’ acceptance of an immersive environment [3].

### 4.4. Micro-Imperfections

The fourth level of the hierarchy concerns micro-imperfections, which refine realism by reducing the artificial appearance of visually perfect surfaces. The reviewed literature consistently indicates that surfaces that appear too smooth or too idealized are often perceived as unnatural. In early realism-classification studies, subjects produced more “real” judgments when viewing non-ideal surfaces, whereas very smooth surfaces reduced perceived realism [5].

Gloss and roughness are not perceived independently. Changes in mesoscale roughness can bias perceived gloss and vice versa, so when these two cues are combined in ways that appear inconsistent, the believability of the overall stimulus decreases even if each component appears individually plausible [14]. More recent work further shows that learned regularities derived from natural image statistics can predict both accurate perceptions and systematic misperceptions of gloss [31].

In rendering practice, micro-imperfections often appear as glints, sparkles, and high-frequency specular variation. Studies of high-resolution normal-mapped and microfacet surfaces have shown that such a microstructure produces characteristic specular responses that contribute significantly to the realistic impression of many materials [32,33,34]. At the same time, the perceived material appearance of a surface depends on its geometry. Even when identical reflectance parameters are used, materials optimized on canonical shapes may not transfer equally well to architectural elements that differ in curvature and scale [34]. This places micro-imperfections below the broader view-dependent layer in Figure 2: they refine realism, but usually after the more dominant cue levels are already in place.

### 4.5. Translucency

The fifth and most context-dependent level of the hierarchy is translucency. Translucency and subsurface scattering form a specialized cue class that is particularly relevant for selected material categories (e.g., marble, onyx, thin plastics), but generally have a narrower influence on realism judgments in architectural scenes than lighting, grounding, or broader material cues.

Rather than depending primarily on accurate recovery of physical material parameters, translucency perception is driven by specific image-level information, especially edge behavior and luminance variation through thin regions [35]. Psychophysical studies show that translucency can systematically affect shape perception. Because translucent surfaces tend to smooth luminance gradients that would otherwise indicate surface relief, they often reduce the perceived amount of shape variation relative to opaque surfaces [36]. Models of material perception relate differences in perceived translucency to phase-function properties and support the existence of a relatively low-dimensional perceptual space in which changes in rendering parameters produce corresponding changes in material appearance [37].

Judgments of translucency are also strongly influenced by lighting direction, which suggests that materials with subsurface components cannot be defined uniformly across contexts through fixed material parameters alone [38]. Mechanisms related to translucent edges help explain why boundaries and thin features are weighted disproportionately in translucency judgments [39]. This line of work further suggests that the physical (shape/material) characteristics of a light-permeable object are interdependent because both rely on the same visual cues, including luminance gradients and specular reflections [40]. Related work on perceived opacity shows that opaque surfaces can sometimes be interpreted as translucent under specific combinations of lighting and gradient structure [41]. For architectural visualization, this means that excessive gradient softening or overly diffuse lighting may unintentionally shift the perceived appearance of a surface toward translucency, thereby undermining the intended material reading.

### 4.6. Workflow Implications

Taken together, the reviewed studies support a layered perceptual structure for architectural visualization workflows. Lighting and grounding cues—especially global illumination and contact shadows—establish the primary framework of scene plausibility [5,6,7,8,9,10,11,12,16]. Once that framework is in place, texture fidelity and consistent material behavior become the main contributors to object-level believability [22,23,24,25]. View-dependent effects become increasingly important during motion, inspection, and immersive presentation, where they function both as material cues and as authenticity signals [15,26,27,28,29,30]. Micro-imperfections further strengthen realism when they remain consistent with the shape and reflectance characteristics of the object being rendered [13,14,31,32,33,34]. Translucency-related cues, by contrast, are more specialized and more prone to context-specific misinterpretation [35,36,37,38,39,40,41].

This layered structure has direct practical implications for rendering prioritization. When computational resources are limited, the largest perceptual benefits are likely to come from first establishing plausible lighting and grounding, then preserving material and texture fidelity, and only afterwards refining the scene through view-dependent effects and surface-level microstructure. This does not mean that lower levels are unimportant, but rather that their contribution depends more strongly on the successful establishment of higher-level plausibility cues.

From a production standpoint, the priority given to texture fidelity and material behavior in Table 2 aligns with practical constraints in texture authoring. For example, work examining Ptex-based texturing shows how the feasibility and efficiency of very high-resolution surface detail can depend on polygon topology and asset structure, which can shape how far texture-driven material believability can be pushed in real projects [42]. Similarly, the AR column in Table 2 highlights that plausibility is not only a rendering issue but also a registration issue: when rendered content is combined with a physical environment, errors in alignment and scene grounding can undermine realism even if material cues are otherwise plausible. Recent AR and LiDAR scanning workflows provide an applied example of these integration constraints in practice [43].

## 5. Limitations

The first of these limitations concerns the separation of individual visual cues into controlled conditions, as opposed to being used in combination. Many psychophysical studies manipulate one factor at a time (e.g., shadow softness, surface roughness, translucency) and keep all else equal [5,6,12,31]. While the manipulation of individual factors is necessary to establish cause-and-effect relationships, it creates a situation in which the results of studies examining individual factors are not representative of how these factors interact in real-world scenarios (i.e., architectural visualization). When cues are used in combination in a more realistic scene, the results from a study in which one factor is manipulated may not linearly increase [18,23,27] when all cues are used in conjunction.

Another limitation includes the reliance upon simplified stimuli in foundational perception research. Studies have been conducted using abstract shapes, grayscale renditions, and canonical objects (e.g., spheres and statues) [10,14,21,31]. Although these types of stimuli are useful for studying basic perceptual processes, they fail to adequately represent the structure and semantics present in architectural environments. Material perception relies heavily upon contextual “stuff” cues that are missing in many laboratory settings [18], limiting the ability to directly apply findings from foundational perception research to full-scale architectural scenes. Accordingly, the conclusions of this review should be interpreted as convergent across methodological traditions, but still bounded by the trade-off between experimental control and ecological realism.

Third, small sample sizes continue to be prevalent, especially in classic psychophysical studies. Several of the most influential studies included fewer than ten participants [10,21,31]. While small sample sizes allow researchers to obtain precise data from within subjects, they severely limit the ability of researchers to generalize their results to larger populations. Larger sample sizes have become more common in VR and AR studies but have often come at the expense of experimental control to provide greater ecological realism [1,7,8,22].

Additionally, a substantial portion of the literature concerning rendering-based techniques provides justification for realism based solely upon perception and does so without providing direct human subject experimental support. Improvements in visual realism (e.g., global illumination, soft shadows, and photon mapping) are often demonstrated through the authors’ own visual inspection alone, resulting in an indirect relationship between physical accuracy and perceived realism [19,25,34,39,40].

Finally, temporal and interactive aspects of realism are underrepresented. Most studies of realism examine it under static viewing or limited motion conditions, even though dynamic walkthroughs, VR, and AR are becoming increasingly relevant to architectural visualization. Fewer than five studies within the reviewed corpus specifically address viewpoint-dependent dynamics, motion parallax, or temporal coherence [1,15,18,38] and therefore limit our ability to draw broad conclusions about realism during active exploration.

## 6. Conclusions

The reviewed literature suggests that perceived photorealism depends less on physical correctness per se than on whether rendered images satisfy learned perceptual expectations.

Across the reviewed literature, the cues most consistently linked to perceived photorealism are lighting cues and grounding cues. Soft contact shadow and coherent cast shadow behaviors have been identified as being particularly influential in providing viewers with visual cues that provide a sense of spatial credibility. Conversely, when soft contact shadows and/or coherent cast shadows are included in an image, the observer’s ratings of photorealism increase substantially regardless of whether any other features of the scene are changed [5,6,7]. Global illumination introduces smooth luminance gradients and interreflections into an image, both of which make it easier for the observer to infer the shapes and spatial relationships contained within the image. Observers have shown a preference for images that contain such indirect lighting structures under practical fidelity constraints in immersive VR environments, which is consistent with a more general account of presence and plausibility in which visual coherence is a primary determinant of whether an individual accepts a virtual environment as being behaviorally credible [3].

A second primary finding of this review is that material believability is usually a function of both texture characteristics and statistical properties of the rendering. Studies of texture and material fidelity indicate that the physical appearance of a rendered object’s surface will generally be judged as more realistic than its geometry if it has a greater degree of visual detail (i.e., texture) and/or a higher level of physically based rendering (PBR). These studies also show that observers can detect fine surface structure even when individual texture elements are not fully resolvable, suggesting reliance on statistical image information rather than only on explicit detail [22,23,24,25]. Perception research also shows that image statistics (the average, variance, etc.) of the surface characteristics of objects are strong predictors of how those surfaces appear and that there are systematic relationships between roughness and gloss, indicating that realism decreases when those two surface characteristics are presented with inconsistent cues [13,14,31]. Research on rendering glints and microstructures has demonstrated that the presence of micro-imperfections provides a significant amount of diagnostic information about the material type (metal, coated finish) and therefore contributes to realism through their unique high-frequency specular behavior [32,33].

While both specular reflections and Fresnel modulation contribute to the appearance of the shape and the type of material, they are also used as authenticity cues during motion because the changes in the viewpoint-contingent image of the reflection match how we perceive visual changes in the world [15,26,27,28,29,30]. In contrast, although material perception does not rely on the geometric shape alone, it does not occur independently of the geometric shape; therefore, material libraries for architecture cannot be optimized using only canonical spherical forms and need to be tested against representative forms [34].

In addition, subsurface scattering and translucency represent a second category of realism cues that have strong dependencies upon context. Both are perceived on the basis of specific aspects of images, most importantly, the behavior of edges and thin regions, and are significantly influenced by the direction of the lighting. Therefore, if used correctly, translucency can increase realism for some materials, such as marble or Onyx; however, if the gradients and edges move to areas of an image where they are indicative of a light-permeable material, they can cause viewers to incorrectly interpret them [35,36,37,38,39]. Further research has shown that viewers can infer both the shape and material characteristics of objects, and there is evidence that viewers can systematically misestimate the translucency-related appearance of materials, depending on the configuration of the gradient in an image; this presents a direct risk to architects: architecturally pleasing and visually smooth lighting conditions can inadvertently cause surfaces to be misinterpreted to indicate unintended material identities [40,41].

In addition to defining how practitioners can best take advantage of these cues, synthesis also highlights areas of opportunity for future research. Specifically, researchers could investigate the relationships among various cues in more complex architectural environments. Additionally, researchers could expand upon previous studies by investigating temporal coherence and active exploration in architectural visualization. Finally, researchers could develop frameworks that integrate perceptual accuracy, subjectively perceived realism, and performance of tasks related to building design and construction within real-world production environments [3,4,18].

Beyond its immediate implications for rendering optimization, the present review also speaks to broader challenges in human-centered visualization: how to allocate limited computational resources, how to design perceptually efficient real-time pipelines for VR/AR and digital twins, and how to evaluate realism in ways that better reflect human judgment rather than physical simulation alone. In this sense, the proposed cue-based taxonomy is intended not only as a summary of existing findings, but also as a framework for future work on perceptually adaptive rendering, cross-modal realism, and evaluation standards for next-generation visualization systems.

## Figures and Tables

**Table 1:** Mapping of dominant perceptual cue categories to their primary perceptual functions, reported effects, and representative studies across the reviewed literature.

Cue Category	Primary Perceptual Function	Key Findings	Representative Studies
Contact shadows	Spatial grounding	Positioning accuracy increased by up to ~45%	[5,6,9,21]
Global illumination	Shape coherence	Supports shape sensitivity	[8,10,16]
Texture and PBR	Material believability	Texture often outweighs geometry	[22,23,24]
View-dependent effects	Material and presence	Supports gloss constancy and presence	[15,29,30]
Micro-imperfections	Statistical realism	Consistent with learned material priors	[13,31,32]
Translucency	Material inference	Shape judgments influenced by edge-based cues	[35,36,37,38,39,40,41]

**Table 2:** Context dependency of perceptual cues in architectural visualization.

Cue	Static Images	VR	AR	Notes
Global illumination	High impact	Very high	Medium	Prioritized under computational constraints
Shadows	Very high	Very high	Critical	Gatekeeper for spatial plausibility
View-dependent effects	Medium	High	High	Strong under motion
Micro -imperfections	Medium	Medium	Low	Material-specific
Translucency	Low–Medium	Medium	Low	Risk of misinterpretation

## Data Availability

No new data were created or analyzed in this study. All information synthesized in this review was derived from previously published sources.
